# Identification of Patterns of Trace Mineral Deficiencies in Dairy and Beef Cattle Herds in Spain

**DOI:** 10.3390/ani15172480

**Published:** 2025-08-23

**Authors:** Candela Fernández-Villa, Lucas Rigueira, Marta López-Alonso, Belén Larrán, Inmaculada Orjales, Carlos Herrero-Latorre, Víctor Pereira, Marta Miranda

**Affiliations:** 1 Department of Anatomy, Animal Production and Clinical Veterinary Sciences, Faculty of Veterinary, University of Santiago de Compostela, Campus Terra, 27002 Lugo, Spain; candela.fernandez.villa@usc.es (C.F.-V.); lucas.rigueira@usc.es (L.R.); inma.orjales@usc.es (I.O.); 2 Rof-Codina Veterinary Teaching Hospital, Faculty of Veterinary, Campus Terra, Universidade de Santiago de Compostela, 27002 Lugo, Spain; 3 Department of Animal Pathology, Faculty of Veterinary, University of Santiago de Compostela, Campus Terra, 27002 Lugo, Spain; marta.lopez.alonso@usc.es (M.L.-A.); belen.larran.franco@usc.es (B.L.); victor.pereira@usc.es (V.P.); 4 Aquatic One Health Research Center (iARCUS), Department of Analytical Chemistry, Nutrition and Bromatology, Faculty of Sciences, Campus Terra, University of Santiago de Compostela, 27002 Lugo, Spain; carlos.herrero@usc.es

**Keywords:** trace minerals, cattle, deficiencies, production systems, selenium, iodine, copper, multielement deficiencies

## Abstract

Microminerals such as cobalt (Co), copper (Cu), iodine (I), iron (Fe), manganese (Mn), molybdenum (Mo), selenium (Se), and zinc (Zn) play key roles in cattle health. However, trace element imbalances are often underdiagnosed. This study retrospectively analyzed serum samples from 1273 cows across 117 herds in Spain, encompassing conventional dairy (*n* = 46), pasture-based dairy (*n* = 11), organic dairy (*n* = 25), and semi-extensive beef (*n* = 35) systems. Trace elements were determined by inductively coupled plasma mass spectrometry (ICP-MS). All herds were investigated for clinical or productive issues where mineral deficiencies were suspected. Significant differences were found in serum trace mineral concentrations between production systems. Adequacy rates were highest in conventional dairy herds receiving routine mineral supplementation, while deficiencies in Se, I, and Cu were frequently detected in pasture-based, organic, and beef herds. Zinc deficiencies were rare and typically involved complex, combined deficiencies. At the farm level, multielement deficiencies (≥3 elements) were detected in 39–45% of organic, pasture-based, and beef herds, but in only 5% of conventional dairy herds (*p* < 0.001). Principal component and cluster analyses produced consistent groupings of minerals according to dietary supplementation and soil-driven exposure. These findings highlight the increased vulnerability of low-input systems to complex micromineral imbalances and underline the importance of system-adapted mineral-monitoring and supplementation strategies in herd health management. However, as the study is based on diagnostic submissions rather than a randomized herd survey, the findings should be interpreted with caution due to potential selection bias.

## Simple Summary

Trace mineral imbalances are often overlooked in cattle herd health investigations despite their potential importance in subclinical disorders affecting reproduction, growth and disease resistance. This study evaluated the prevalence and patterns of micromineral deficiencies in cattle herds in Spain using diagnostic samples submitted for unresolved clinical or production problems. The occurrence of deficiencies varied markedly across production systems, and the prevalence of multielement imbalances was highest in organic, pasture-raised dairy, and semi-extensive beef herds. The findings emphasize the need for comprehensive monitoring of mineral status in low-input and semi-extensive cattle-farming systems.

## 1. Introduction

Microminerals such as selenium (Se), copper (Cu), zinc (Zn), and iodine (I) are essential nutrients for all livestock species, as they support key physiological functions including immunity, reproduction, growth, and antioxidant defense [1,2,3,4,5]. In monogastric species such as pigs and poultry, overt deficiencies are relatively uncommon due to the use of compound feed with precisely formulated and uniformly distributed mineral supplementation. By contrast, ruminant diets are typically composed of varying proportions of pasture, conserved forage, and concentrates, depending on the type of production system. Since mineral supplementation is generally only incorporated in the concentrate portion (which often represents a small fraction of the total ration), the overall micromineral intake may be insufficient, particularly when the forage originates from mineral-deficient soils [6,7,8,9]. As a result, both clinical and in particular subclinical deficiencies may develop, potentially compromising animal performance, fertility, and disease resistance [1,10,11,12,13]. While classical clinical signs such as nutritional muscular dystrophy (Se), enzootic ataxia (Cu), goitre (I), and parakeratosis (Zn) can occur in severe cases, most deficiencies present with non-specific signs that often go unnoticed in routine herd health monitoring [2,3].

Micromineral imbalances are frequently overlooked in routine veterinary investigations, as on-farm diagnostic efforts tend to focus on infectious and parasitic diseases, major dietary imbalances related to energy and protein, and common metabolic disorders [2,10]. Trace element status is often considered only after these more prevalent causes have been excluded [4,10,14], typically as a last step in the diagnostic process. As a result, mineral testing is typically delayed until clinical signs persist or performance issues become evident, by which time the herd may already be suffering serious losses due to impaired growth, reduced fertility, and/or increased disease susceptibility. This reactive diagnostic approach probably contributes to the systematic underestimation of both the prevalence and the impact of trace mineral deficiencies in cattle.

Cattle production systems in Spain are diverse, encompassing conventional indoor dairy farms, pasture-based dairy operations, organic dairy systems, and extensive beef herds. These systems differ markedly in feeding practices, particularly in the proportion of concentrate in the diet and the extent to which mineral supplementation is applied [8,15]. Indoor dairy farms tend to rely more heavily on mineral-fortified concentrate feed, whereas pasture-based, organic, and extensive systems obtain a larger share of nutrients from forage and are often subject to economic or regulatory restrictions regarding supplementation. In addition, the geological characteristics of soils in many areas of Spain contribute to naturally low concentrations of certain trace elements, particularly Se and Cu, and to a lesser extent I, which can result in poor micronutrient content in locally produced forage [8]. As a result, the risk of micromineral deficiency may vary substantially across systems, both due to differences in dietary structure and to the mineral composition of available feed resources. However, comparative data on trace element status in cattle across these production systems remain scarce [8,9] despite the potential implications for animal health and productivity.

Our diagnostic laboratory receives blood, serum, and tissue samples from cattle farms across Spain as part of veterinary investigations into unresolved clinical or productive problems. In most cases, the samples are submitted after common infectious, metabolic, and nutritional causes have been ruled out, and mineral imbalances are thus suspected as a possible underlying factor. Although the dataset does not originate from a structured epidemiological survey, it reflects real-world conditions in commercial herds where clinical signs have prompted further investigation. This retrospective sample collection thus provides a valuable opportunity to explore the occurrence of trace mineral deficiencies in a variety of production systems and geographic areas under practical field conditions. However, it is important to acknowledge that the samples were not randomly selected but originated primarily from herds with suspected clinical or production issues, which may introduce a selection bias toward problematic herds. By analyzing patterns across these submissions, it is possible to identify system-specific vulnerability profiles and generate hypotheses for targeted monitoring and prevention strategies.

The aim of this study was to describe the frequency and type of micromineral deficiencies identified in diagnostic samples from cattle herds in Spain, and to assess how their distribution varies across four production systems: conventional indoor dairy, pasture-based dairy, organic dairy, and extensive beef systems. The findings may help to raise awareness of the role of trace element status in herd health problems and support more proactive, system-adapted supplementation and diagnostic strategies in the field.

## 2. Materials and Methods

### 2.1. Study Design and Sample Selection

This retrospective study was based on serum samples submitted for trace mineral analysis to the IMedA Research Group laboratory at the Faculty of Veterinary of the University of Santiago de Compostela (USC) (Lugo, Spain), between 2016 and 2024. Samples originated from cattle farms located in various regions of Spain and were submitted as part of veterinary investigations into clinical or productive problems.

Herds were eligible for inclusion if complete information, based on the information provided by the submitting veterinarian, on production system, nutritional management, and health status was available. A minimum of seven animals per farm was required, except for beef cattle farms, where the threshold was lowered to five due to smaller herd sizes. Furthermore, only those farms on which all essential trace minerals, i.e., cobalt (Co), copper (Cu), iodine (I), iron (Fe), manganese (Mn), molybdenum (Mo), selenium (Se), and zinc (Zn), had been analyzed were considered. As an exclusion criterion, herds that had received targeted trace mineral supplementation (oral boluses, drenches, or parenteral treatments) prior to sampling were not included, in order to minimize bias due to previous interventions.

Data on the clinical reason for each diagnostic request (e.g., reproductive disorders, growth retardation, among others) were collected and are presented in Table 1. The final dataset included a total of 1273 animals from 117 herds, distributed as follows: 466 animals from 46 conventional indoor dairy herds, 120 animals from 11 pasture-raised dairy herds, 464 animals from 25 organic dairy herds, and 223 animals from 35 semi-extensive beef herds. Conventional dairy herds were housed indoors and fed total mixed rations tailored to production requirements. Pasture-based dairy cows obtained part of their dry matter intake from grazing, combined with varying levels of concentrate supplementation. Organic dairy herds, managed according to organic farming regulations, consumed a higher proportion of dry matter as forage. Semi-extensive beef cattle were reared on exclusively forage-based diets, without concentrate supplementation.

### 2.2. Trace Mineral Analysis and Quality Assurance

Serum samples were subjected to acid digestion (all trace minerals except I) and alkaline extraction (I) prior to inductively coupled plasma mass spectrometry (ICP-MS) analysis, as previously described [16].

Briefly, the serum samples (1 mL) were digested by mixing with 1 mL of hyper-pure nitric acid (69%) and 0.5 mL of hydrogen peroxide (33%) in propylene tubes, which were then held at 60 °C for at least 2 h in a thermostatic block. The digested samples were diluted with 2.5 mL of Milli-Q ultrapure water. For determination of total I, samples were processed by a high-temperature alkaline extraction procedure with a mixture of tetramethylammonium hydroxide 25% (*w*/*v*) in water. Concentrations of essential trace minerals (Co, Cu, Fe, I, Mn, Mo, Se, and Zn) were quantified using ICP-MS (Agilent 7700×ICP-MS system; Agilent Technologies, Tokyo, Japan), in the Research Infrastructures Unit of the USC (Lugo, Spain). This laboratory operates under a stringent analytical quality control protocol and holds ISO accreditation, ensuring the reliability and traceability of the results.

An analytical quality control program was applied throughout the analysis. Blank samples were processed during the analysis, and the readings obtained were subtracted from the sample readings to produce the final values. The limit of detection (LOD) was calculated as 3 times the standard deviation of the blanks. The concentrations were above the LOD in all samples. The accuracy of the determinations was verified by measuring certified reference material provided by NIST (Animal serum 1598a) and in-house reference cattle serum. Overall, good recoveries were achieved (ranging from 88 to 113%).

### 2.3. Statistical Analysis

Descriptive statistics for serum trace mineral concentrations were calculated as mean, standard deviation (SD), median, and range. Differences among production systems were assessed using one-way analysis of variance (ANOVA), followed by Tukey’s post hoc test for multiple comparisons. Prior to applying ANOVA, data were tested for normality using the Shapiro–Wilk test and for homogeneity of variances using Levene’s test. The prevalence of farm-level deficiency was also compared in order to identify differences in the number of deficient elements per herd across production systems. For this purpose, herds were categorized according to the number of deficient elements identified (0, 1, 2, or ≥3 elements), and Chi-square tests were used to identify differences between production systems. When expected cell counts were below 5, Fisher’s exact test was applied.

Multivariate analysis was used to assess overall patterns in trace mineral profiles across production systems. Principal component analysis (PCA) was used to explore the relationships between samples and variables and to reveal the latent structure of the information contained in the data matrix (X_1273×8_), and was performed using standardized serum concentrations of all trace elements. The first three principal components were retained on the basis of the proportion of variance explained and scree plot evaluation. Hierarchical cluster analysis (HCA) was subsequently applied within each production system to explore associations between trace elements, using Ward’s linkage method and Euclidean distance.

All statistical analyses were conducted using SPSS Statistics version 28.0 (IBM Corp., Armonk, NY, USA). Statistical significance was set at *p* < 0.05.

## 3. Results and Discussion

### 3.1. Trace Mineral Status in Cattle According to Production System

The serum trace element concentrations of the cattle included in this study, stratified by production system, are summarized in Table 2.

The Se, I, and Cu levels indicated deficiencies, and Zn levels indicated marginal imbalances. Among these trace minerals, Se deficiency is of particular concern given the extent of the observed deficits. Only conventional dairy farms exhibited adequate mean serum concentrations (79.2 ± 16.6 µg/L), while pasture-raised (47.6 ± 28.1 µg/L), organic (47.1 ± 29.0 µg/L), and beef herds (34.3 ± 21.5 µg/L) all remained below the established adequacy threshold of 65 µg/L [17], reflecting widespread suboptimal Se status. In some areas of Spain and Europe, Se concentrations in soils are generally low, making Se deficiency one of the most common and important trace element disorders in ruminants [8,9,10,11,15,18,19]. Selenium is routinely incorporated in concentrate feed to ensure adequate intake and prevent deficiency, especially in conventional dairy systems where concentrates are more frequently used [3,8]. However, in systems where concentrate feeding is less common, such as organic and pasture-based dairy farms, serum Se concentrations are frequently suboptimal, although generally not as severely reduced as in beef herds. In beef farm systems, in which concentrate supplementation is minimal or absent, Se levels are often very low [7,9], and Se deficiency is the most common mineral deficiency in grazing animals [6]. Particular attention is therefore needed to ensure appropriate Se intake in all systems with limited concentrate use, in order to prevent clinical and subclinical deficiencies.

Iodine followed a similar pattern to Se. Adequate mean serum I concentrations (98.0 ± 62.9 µg/L) were only detected in conventional dairy herds, while levels were consistently lower in pasture-raised (65.9 ± 45.0 µg/L), organic (62.0 ± 63.9 µg/L), and beef herds (62.6 ± 29.5 µg/L), with a substantial proportion of animals falling below the recommended threshold of 80 µg/L [17]. As observed for Se, these differences are closely related to feeding practices, since I is routinely included in concentrate formulations used in conventional dairy systems but is much less frequently supplemented in organic, pasture-raised, and beef herds [19]. In addition, I concentrations in soils across Spain are naturally low due to the geochemical characteristics of the soils. Furthermore, the presence of goitrogenic substances in Brassica spp. (turnips, rapeseed, kale) and white clover forage resulted in a widespread risk of dietary I deficiency [2,3,19].

Copper concentrations were also reduced, particularly in organic (0.642 ± 0.154 mg/L) and beef herds (0.592 ± 0.180 mg/L), with a high proportion of animals/farms showing serum levels below the critical threshold of 0.6 mg/L [17]. Copper deficiency remains one of the most common trace mineral disorders in grazing cattle worldwide [5]. These lower concentrations reflect the low Cu content in the soil in some areas of Spain and the limited Cu supplementation typically applied in extensive and organic systems, and they also indicate the complex metabolic regulation of Cu in ruminants. Unlike other trace elements, Cu status is not solely determined by dietary intake and is also strongly influenced by the presence of dietary antagonists. In particular, Mo, Fe, and sulfur (S) interact with Cu in the rumen, forming insoluble complexes that reduce its bioavailability and intestinal absorption [2,5,20]. Correct evaluation of Cu adequacy in ruminant diets must therefore consider both Cu concentrations and the levels of these interacting elements.

Regarding Zn, no statistically significant differences were observed between production systems, with mean serum concentrations ranging from 0.859 to 0.883 mg/L across all groups. Marginally deficient Zn serum concentrations were only detected in a single extensively managed beef herd, in which some animals had serum Zn concentrations below 0.6 mg/L [17]. Therefore, Zn deficiency does not appear to be a widespread problem in the studied populations, but rather an occasional issue limited to isolated herds.

Cobalt, Fe, Mn, and Mo serum concentrations were within normal reference ranges [17], and therefore not suggestive of clinical deficiencies, antagonisms, or imbalances. However, significant differences between production systems were observed. Overall, extensive beef cattle exhibited significantly lower serum concentrations of Co, Mn, and Mo, along with higher Fe levels than in dairy herds. These differences probably reflect the combined effects of the pasture-based management system and the specific environmental conditions of the grazing areas. The minimal use of mineral supplementation in extensive systems, particularly regarding Co and Mn, which are routinely provided through concentrate premixes in dairy herds, contributes to the lower levels of these two microminerals [2]. By contrast, the elevated Fe concentrations commonly observed in beef herds may result from ingestion of soil during grazing, a well-documented phenomenon in extensively managed ruminants [21]. Regarding Mo, the low levels in beef cattle probably reflect the geographic variability of soil content [18]; many of these farms are located in areas where the soil is naturally poor in Mo [2,18].

### 3.2. Farm-Level Trace Mineral Deficiency Patterns in Different Production Systems and Clinical Reason for Submission

**Figure 1:**
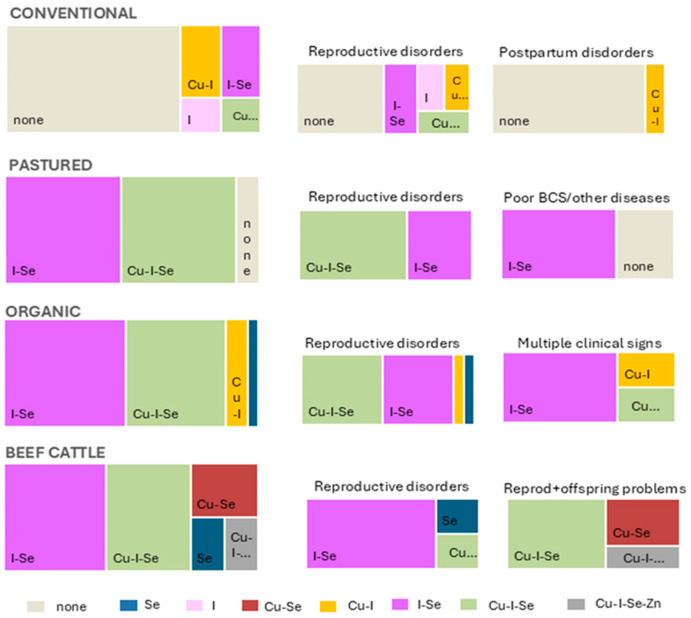
Percentage of farms with mineral deficiencies, classified according to the number of deficient elements detected. Data are presented for each production system (left-hand side) and subdivided by clinical category at submission (right-hand side), including reproductive disorders, postpartum disorders, low body condition score (BCS), or other diseases, multiple clinical signs, and offspring-related disorders.

The distribution of trace mineral deficiencies at the herd level, categorized according to the number of deficient elements identified, is summarized Figure 1 and Table 3.

The distribution of trace mineral deficiencies differed significantly across production systems (Chi-square = 44.53; df = 9; *p* < 0.001). Specifically, deficiencies affecting three or more elements were identified in 45.5% of dairy pasture-raised herds, 39.1% of organic dairy herds, and 40.0% of beef herds, but in only 5.3% of conventional dairy farms.

The prevalence and complexity of deficiencies were lowest in conventional dairy herds, with 68.4% of herds showing no deficiencies and only isolated cases of Cu and I imbalances detected in postpartum cows. Even among conventional herds referred for reproductive problems, deficiencies were generally limited to one or two elements, suggesting that the regular use of mineral-fortified concentrates in these systems provides effective coverage for most trace elements [3,8].

By contrast, the prevalence of multielement deficiencies was much higher in pasture-raised dairy, organic dairy, and beef herds, and often involved combinations of Se, I, and Cu deficiencies. This complexity was particularly evident in beef herds, where deficiencies affecting multiple elements frequently associated with reproductive disorders and neonatal problems. Similarly, multielement deficiencies were frequent in organic dairy herds, particularly in farm systems with reproductive problems, while pasture-based systems occupied an intermediate position with patterns resembling those on organic farms. It should be noted that differences in veterinary access and diagnostic practices across production systems may influence the detection and reporting of these mineral deficiencies, which could impact the observed patterns.

**Figure 2:**
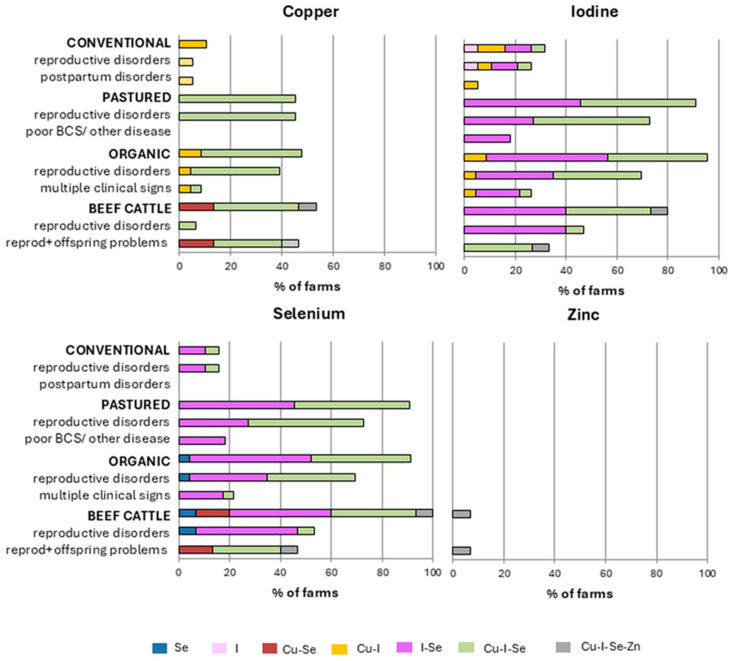
Distribution of mineral deficiencies by element and combination across production systems and clinical categories. The first bar for each production system represents the total number of herds within that system. The other bars correspond to clinical subgroups within the same production system (e.g., reproduction and postpartum for conventional dairy), which together represent the total number of farms in that system. Deficiencies are classified according to the specific elements involved, either as isolated deficiencies or as combined deficiencies involving copper (Cu), selenium (Se), iodine (I), and zinc (Zn).

Figure 2 shows the specific elements involved in mineral deficiencies across the different production systems and clinical categories.

Overall, I and Se were the most frequently affected elements, with more than 80% of the dairy farms in pasture-based, organic, and semi-extensive beef systems presenting deficiencies involving these two elements. However, isolated deficiencies in I or Se were uncommon; in most cases, these elements were simultaneously deficient (I-Se) or involved in more complex deficiency patterns involving additional trace elements. Selenium plays a crucial role in I metabolism, particularly through its involvement in the synthesis and activation of thyroid hormones. As a component of selenoenzymes such as iodothyronine deiodinases, Se is essential for the conversion of thyroxine (T4) into its active form, triiodothyronine (T3) [2]. Therefore, Se deficiency can exacerbate the effects of I deficiency, impairing thyroid function even when I intake is marginally adequate.

Cu deficiencies were less frequent, affecting up to 50% of the farms depending on the production system, particularly organic dairy and beef systems. Notably, Cu deficiency was never observed in isolation but always occurred in combination, predominantly with I and Se (e.g., Cu-I, Cu-I-Se). Finally, as previously mentioned, Zn deficiencies were the least common, occurring in less than 10% of the beef herds, exclusively as part of complex combined deficiencies involving four trace elements.

Enjalbert et al. [11] reported that Se and Cu deficiencies are significant risk factors for impaired productivity, fertility, and overall health in both dairy and beef cattle in France. Likewise, Guyot et al. [10] found that trace element deficiencies were associated with a higher incidence of various pathologies in Belgian herds, often involving multiple elements and multiple clinical conditions within the same farms. A recent study by Mitchell et al. [12] analyzed 251 cases of abortions, stillbirths, and early neonatal deaths in beef cattle herds from the upper Midwest (USA), revealing a significant association between low copper and high manganese levels in fetal liver and increased risk of infectious abortions in beef cattle. Poor fertility can be associated with multiple trace mineral imbalances, mainly Se, I, and Cu, but Se imbalance is the most important in reproductive problems. Herds with marginal status of Se have enhanced risk of abortion, perinatal mortality, reproductive failure, metritis, and retained placenta [11]. Moreover, Se deficiency can promote I deficiency, which is known to induce abortion, stillbirth, and growth retardation [2]. In the present study, Se and I were the most commonly deficient elements in all types of farming; this could be associated with the high percentage of herds with reproductive problems. Lower contents of Zn, Cu, I, and Se in serum were also related with mastitis [22] and lameness [23].

In the present study, beef cattle herds with concurrent deficiencies in Cu, Se, and/or I were those in which health problems in the offspring were most frequently observed. This may be associated with impaired immune function in both dams and calves, as Cu plays a critical role in immunity through its involvement in numerous enzymatic systems [1,2]. Similarly, Enjalbert et al. [11] reported that low plasma Cu concentrations in cows were linked to an increased risk of health disorders and growth retardation in their calves.

Our findings reflect the vulnerability of low-input and extensive production systems to complex trace element imbalances. In these systems, the reduced use of concentrate feeds and mineral supplements, together with the naturally low trace mineral content of soils and forage in many areas of Spain, contributes to the occurrence of multielement deficiencies. The frequent association of these imbalances with reproductive disorders and offspring problems further highlights their potential clinical relevance. Therefore, comprehensive monitoring of mineral profiles should be considered in routine herd health programs, particularly in systems relying predominantly on pasture and forage.

### 3.3. Multivariate Analysis of Trace Mineral Profiles and Deficiency Patterns

**Figure 3:**
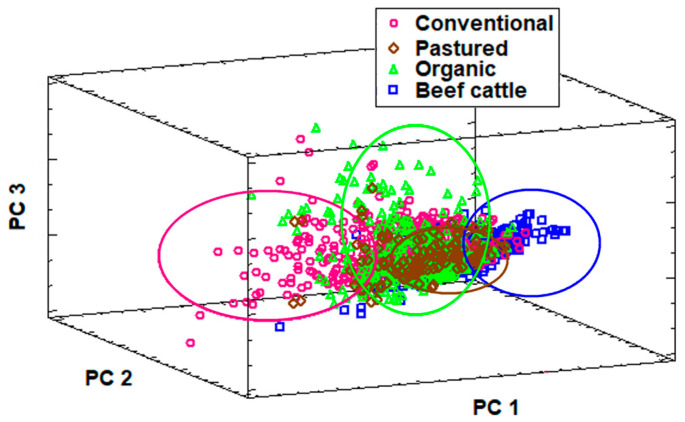
Principal component analysis (PCA) score plot of the serum samples according to type of farm defined for the three principal components representing 54.6% of the total variance.

Principal component analysis was used to assess global differences in trace mineral profiles across production systems (Figure 3). The first three PCs explained 54.6% of the total variance with eigenvalues >1, indicating high contents of information. The PCA plot showed a clear separation between beef cattle herds and conventional dairy herds, which formed two well-defined and distinct clusters.

By contrast, pasture-raised and organic dairy herds occupied an intermediate position, with a considerable degree of overlap between them. This distribution is consistent with the intrinsic characteristics of these production systems. Beef and conventional dairy systems are relatively homogeneous, with beef herds being managed under semi-extensive grazing conditions with minimal supplementation, and conventional dairy herds typically receiving consistent mineral supplementation through balanced concentrate feeds as total mixed ration. By contrast, pasture-based and organic dairy farms displayed greater variability in trace mineral nutrition, particularly in organic systems. This variability is primarily driven by differences in grazing intensity, forage composition, concentrate feeding, and the use of permitted mineral supplements, which depend on individual farm management decisions within the framework of organic certification standards [8,19].

**Figure 4:**
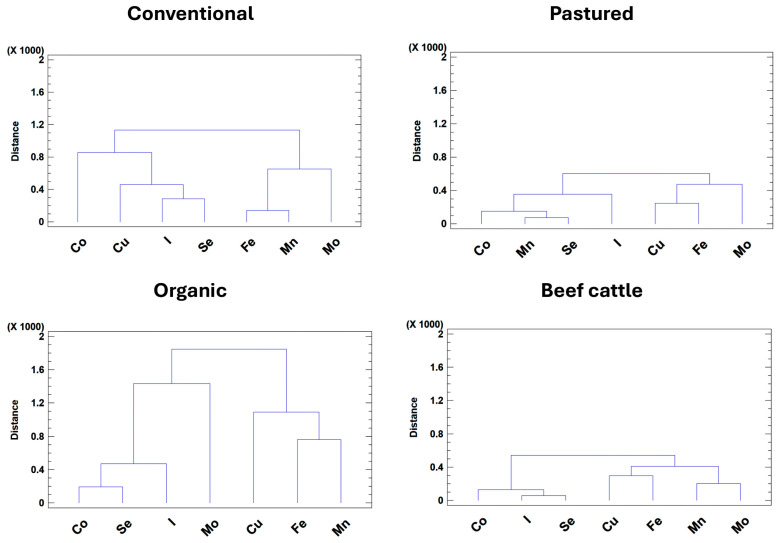
Cluster analysis of trace element associations under different cattle production systems.

Hierarchical cluster analysis (HCA), based of the squared Euclidean distance and using the Ward method as agglomeration procedure, was performed separately for each production system to explore the associations between trace elements under different management conditions (Figure 4).

This analysis revealed a consistent structure across all production systems, with two clearly distinguishable major clusters of trace elements. The first cluster corresponded to “nutritional inputs”, including Co, Cu, I, and Se, which are primarily supplied through concentrate feed and mineral supplements [2,3,8,14]. The second cluster represented “soil-driven elements”, composed of Fe, Mn, and Mo, the concentrations of which largely depend on soil mineral composition, forage uptake, and, in some cases, direct ingestion of soil during grazing [2,18,21]. These clustering patterns matched biological expectations and are consistent with previous findings in cattle reared in different production systems, highlighting the combined influence of dietary management and environmental exposure on trace mineral status in ruminants [2,6,7,8]. While this general clustering pattern was preserved in all systems, differences in the strength and stability of these associations emerged across production types. In pasture-raised dairy and beef herds, in which supplementation is reduced and the levels predominantly depend on input from pasture, Cu was more strongly associated with its antagonists (Fe and Mo). This underscores the importance of considering these interactions when evaluating Cu status, as they directly influence Cu bioavailability and utilization in grazing ruminants [2,20].

The degree of dispersion also varied between systems. Organic dairy herds exhibited the highest variability, reflecting their greater heterogeneity in feeding management, concentrate use, grazing intensity, and mineral supplementation practices, all within the framework of organic production standards. This greater variability may be influenced by farm size and geographic location, which can impact nutritional management practices within the constraints of uniform organic certification standards. By contrast, pasture-raised dairy and beef herds presented more homogeneous profiles, probably due to their strong reliance on stable forage-based diets and consistent soil mineral inputs. Overall, these findings reinforce that both feeding strategies and environmental conditions play a critical role in shaping trace mineral profiles across different cattle production systems.

This study has several limitations inherent to its retrospective design. The data were obtained from serum samples submitted to a diagnostic laboratory over an eight-year period, primarily from herds experiencing unresolved clinical or productive issues. As such, the sampling was not randomized or representative of the broader cattle population in Spain, and selection bias is likely. Additionally, due to the diagnostic nature of the submissions, detailed animal-level data—such as age, parity, lactation stage, or precise feeding and supplementation practices—were not consistently available. These factors limit the extent to which causal inferences can be drawn and reduce the generalizability of the results. Therefore, the associations reported here should be interpreted with caution, and further prospective, controlled studies are warranted to validate and expand upon these findings.

## 4. Conclusions

Trace element deficiencies, primarily involving Se, I, and Cu, are common in both dairy and beef herds in Spain. This study highlights the critical role of trace mineral imbalances as underlying contributors to herd health issues, particularly in cases where infectious and major metabolic causes have been ruled out. The occurrence and complexity of these deficiencies were closely linked to the production system, with the highest prevalence found in dairy pasture-raised, organic dairy, and semi-extensive beef herds, where mineral supplementation is often limited or absent. In these contexts, multiple trace minerals (usually Se, I, and Cu) were frequently deficient simultaneously, reflecting limited dietary supply and antagonistic interactions, especially under grazing conditions.

Based on these findings, immediate intervention is most urgently required in pasture-raised dairy, organic dairy, and semi-extensive beef systems, where mineral deficiencies are most prevalent and complex. Practitioners should prioritize regular and comprehensive trace mineral profiling in these herds to enable early detection and targeted supplementation.

Given this scenario, trace mineral assessment emerges as a critical diagnostic tool in herd health investigations, particularly when addressing non-specific reproductive or productive disorders. However, evaluations should not rely solely on individual element measurements. A full profile assessment, considering the production system characteristics, feeding practices, and antagonistic factors influencing bioavailability, is essential. This preventive approach is particularly important in low-input systems, where supplementation is often limited, emphasizing the need for proactive rather than reactive diagnostics. Implementing system-adapted supplementation plans based on these comprehensive assessments will help prevent both clinical and subclinical consequences, improving overall herd health and productivity.

It is important to note, however, that the retrospective nature of the study and the diagnostic origin of the samples may limit the generalizability of the findings.

## Figures and Tables

**Table 1:** Description of farms and serum samples included in the study, categorized by production system and clinical reason for submission.

Production System	No. Farms	Total no. Animals Sampled	Main Clinical Reason for Submission
Conventional dairy	46	466	Reproductive disorders (*n* = 24) Postpartum disorders (*n* = 22)
Pasture-based dairy	11	120	Reproductive disorders (*n* = 8) Poor BCS ^1^/other diseases (*n* = 3)
Organic dairy	25	464	Reproductive disorders (*n* = 19) Multiple clinical signs (*n* = 6)
Beef cattle	35	223	Reproductive disorders (*n* = 18) Reproductive and offspring problems (*n* = 17)
Total	117	1273	

^1^ BCS = body condition score.

**Table 2:** Serum concentrations of trace elements (mean ± SD; median; range) in cattle according to production system.

		Type of Farm				
		Conventional Dairy	Pasture-Based Dairy	Organic Dairy	Beef Cattle	*p*-Value
Co (µg/L)	Mean ± SD	0.776 ± 0.748 ^b^	1.031 ± 0.489 ^a^	1.147 ± 0.424 ^a^	0.270 ± 0.328 ^c^	<0.001
	Median	0.748	1.051	1.024	0.246	
	(min–max)	(0.029–3.390)	(0.376–1.984)	(0.401–3.426)	(0.024–0.922)	
Cu (mg/L)	Mean ± SD	0.721 ± 0.128 ^a^	0.712 ± 0.194 ^a^	0.642 ± 0.154 ^b^	0.592 ± 0.180 ^b^	<0.001
	Median	0.720	0.675	0.636	0.583	
	(min–max)	(0.392–1.410)	(0.381–1.367)	(0.134–1.162)	(0.291–0.927)	<0.001
Fe (mg/L)	Mean ± SD	2.20 ± 0.63 ^ab^	1.81 ± 0.76 ^c^	1.98 ± 0.77 ^cb^	2.27 ± 0.83 ^a^	
	Median	2.17	1.61	1.82	2.13	
	(min–max)	(0.56–4.44)	(0.66–5.04)	(0.17–5.04)	(1.32–3.90)	
I (µg/L)	Mean ± SD	98.0 ± 62.9 ^a^	65.9 ± 45.0 ^b^	62.0 ± 63.9 ^b^	62.6 ± 29.5 ^b^	<0.001
	Median	85.3	62.4	52.7	47.7	
	(min–max)	(14.9–490)	(19.8–196)	(2.4–468)	(15.7–498)	
Mn (µg/L)	Mean ± SD	3.97 ± 1.48 ^a^	3.18 ± 1.52 ^b^	3.72 ± 2.04 ^a^	3.14 ± 1.08 ^b^	<0.001
	Median	3.61	3.08	3.44	2.95	
	(min–max)	(1.66–22.59)	(1.44–6.57)	(1.15–10.3)	(0.89–9.96)	
Mo (µg/L)	Mean ± SD	21.1 ± 9.33 ^b^	18.0 ± 30.5 ^b^	29.7 ± 22.8 ^a^	8.9 ± 14.5 ^c^	<0.001
	Median	14.4	12.1	21.1	5.3	
	(min–max)	(1.7–170)	(2.4–76.1)	(1.5–353)	(0.8–34.0)	
Se (µg/L)	Mean ± SD	79.2 ± 16.6 ^a^	47.6 ± 28.1 ^b^	47.1 ± 29.0 ^b^	34.3 ± 21.5 ^c^	<0.001
	Median	84.2	48.0	42.4	33.7	
	(min–max)	(19.8–141)	(11.2–99.1)	(7.2–133)	(6.6–80.7)	
Zn (mg/L)	Mean ± SD	0.875 ± 0.210	0.859 ± 0.228	0.866 ± 0.202	0.883 ± 0.246	0.399
	Median	0.862	0.805	0.841	0.843	
	(min–max)	(0.360–1.672)	(0.306–1.798)	(0.293–1.829)	(0.236–1.614)	

Different letters indicate statistically significant differences between groups (*p* < 0.05).

**Table 3:** Distribution of farms according to the number of trace element deficiencies identified, categorized by production system.

	0 Elements	1 Element	2 Elements	≥3 Elements
Conventional dairy	32 (68.4%)	7 (15.8%)	5 (10.5%)	2 (5.3%)
Pasture-based dairy	1 (9.1%)	0 (0%)	5 (45.5%)	5 (45.5%)
Organic dairy	0 (0%)	1 (4.3%)	14 (56.5%)	10 (39.1%)
Beef	0 (0%)	2 (6.7%)	19 (53.3%)	14 (40.0%)

## Data Availability

The data that support the study findings are available from the last author upon request.
